# New Drugs and Preparations

**Published:** 1894-02-17

**Authors:** 


### NEW DRUGS AND PREPARATIONS.

[We shall be glad to receive, at our Office, 428, Strand, London, W.C., from the manufacturers, specimens of all new preparations and drugs which may be brought out from time to time.]

### MOYRAPUAMA.

Moyrapuama, a remedy almost unknown on this side of the Atlantic, has been brought to the notice of European readers by Dr. Rebourgeon (*Les Nouveaux Remèdes*, Jan. 24th, 1894), who writes that the therapeutic uses of this plant is known throughout Brazil, where it is held in high reputation as a general nervine tonic and an active aphrodisiac. It is indigenous to the south and south-west regions of the Para State, an almost leafless shrub, growing to the height of about six feet. The earliest references to it are found in the religious writings of the Portuguese and Spaniards, who were sent to convert the inhabitants about 1704. Endowed with therapeutical properties, which have made it a popular panacea, it would have occupied an important place in the Brazilian pharmacopœia if the difficulties in obtaining it had not been, up to the present time, almost insurmountable. No complete scientific investigation of its properties has yet been made, the practitioners who have employed it having been guided mainly by the empirical practice of the Indians as regards its therapeutical virtues. It is said to be non-poisonous. The writer refers to the thesis of Kleesattel[1], of Stuttgart, and Malcher, who differ in placing the plant among the oleaceæ and acanthaceæ respectively. The investigations of C. Rebourgeon have shown that the glucoside, which has been separated in the form of a white powder, is the active principle, and forms a considerable percentage of the powdered root. The physiological experiments have demonstrated that it is remarkably feeble in toxicity, for even so large a dose as a gramme to the kilogramme injected into the veins of dogs, cats, guinea pigs and rabbits did not produce a fatal effect, although each gramme injected represented almost a centigramme of glucoside. But the mucous membranes of the gastro intestinal tract were intensely injected. The heart and aosta showed ecchymosis especially on the valves. The genital organs were slightly congested, as also were the nerve centres. Pregnant animals at once aborted after the injection of a toxic dose. In conclusion, Rebourgeon considers that moyrapuama is indicated in the treatment of neurasthenia in general, and more particularly in asthenic digestive and circulatory disorders, in uterine asthenia, and in genital debility.

[1] Pharmakaguosie der Muira Ruama. Ulm. 1892. [2] Estatistica das arvores Silvestras. Para. 1881.

### DERMATOL.

Which appears to be a neutral Salt of Bismuth, is a preparation of Messrs. Burroughs and Wellcome. It is a fine yellowish powder with a delicate faint smell, almost impalpable in consistency. We have been employing it lately for a very extensive burn of the second and third degree, with much sloughing of the skin. During the early weeks of treatment—before the sloughs all separated—it formed an admirable dressing, being dusted on thickly from a dredger and simply covered with oil-silk. On being washed off for re-application it left a nice healthy granulating surface. We found that it was quite antiseptic, in fact grafting was begun successfully during its use. It makes an excellent powder for "eczema intertrigo."

